# Optimization strategies for care‑giving behaviors of family caregivers for patients with enterostomy: An evidence summary

**DOI:** 10.1371/journal.pone.0356569

**Published:** 2026-09-10

**Authors:** Yujie Liu, Yuhui Zhang, Aiping Li, Guangqin Yun, Xiangyang Chen

**Affiliations:** 1 Nursing School, Inner Mongolia Medical University, Hohhot, Inner Mongolia, China; 2 Nursing Department, The Affiliated Hospital of Inner Mongolia Medical University, Hohhot, Inner Mongolia, China; 3 Gastrointestinal Surgery Department, The Affiliated Hospital of Inner Mongolia Medical University, Hohhot, Inner Mongolia, China; Shuguang Hospital, CHINA

## Abstract

**Background:**

To systematically retrieve and summarize evidence on optimization strategies for caregiving behaviors of family caregivers of patients with enterostomy, and to provide an evidence‑based reference for clinical practice.

**Methods:**

This is an evidence summary that adopts the rigorous methodological framework of systematic reviews. Evidence was searched from databases and websites including UpToDate, BMJ Best Practice, the International Guideline Network, the Agency for Healthcare Research and Quality (USA), the Registered Nurses’ Association of Ontario (Canada), the World Council of Enterostomal Therapists, the Wound, Ostomy and Continence Nurses Society (USA), the Chinese Medical Journal Database, Cochrane Library, Embase, PubMed, and CINAHL. The evidence types included clinical decisions, guidelines, systematic reviews, evidence summaries, and expert consensuses. The search period covered from database inception to January 31, 2026. Literature quality assessment was independently performed by at least two researchers trained in systematic evidence‑based methods. Evidence extraction and synthesis were conducted on the literature that met quality criteria, in combination with professional opinions.

**Results:**

A total of 13 articles were included: 2 clinical decisions, 8 guidelines, 1 systematic review, and 2 expert consensuses. Sixteen pieces of evidence were summarized across four domains: structured empowerment of care knowledge and skills, support strategies for the care process, maintenance of caregivers’ physical and mental health, and construction of a multi‑dimensional support system.

**Conclusion:**

The best evidence summarized in this study is scientifically sound and practical to a certain extent, providing a reliable basis for healthcare professionals to develop intervention plans and implement evidence‑based practice.

## 1. Introduction

Colorectal cancer (CRC) is one of the most common malignant tumors worldwide, ranking third globally and second in China in terms of incidence [1]. Currently, the main treatment for CRC remains comprehensive therapy centered on surgery. Studies have shown that 50%–60% of patients with colorectal cancer require colostomy surgery [2,3]. In China, the number of enterostomy patients has exceeded one million, with approximately 100,000 new cases each year, showing a rapidly increasing trend [4].

The rehabilitation process after enterostomy is complex and prolonged, requiring long‑term professional nursing support. Family caregivers (FCs) play an indispensable role in this process. FCs refer to family members who provide care and support for individuals with chronic diseases, disabilities, or self‑care deficits, including mainly spouses, children, or siblings [5]. Research indicates that active participation by FCs can not only significantly improve patients’ physical function but also effectively relieve their psychological stress and promote the recovery of social adaptability [6,7].

However, due to the highly specialized nature of enterostomy care and the general lack of systematic training and guidance for caregivers, they face numerous challenges in the actual care process [8]. Therefore, this study systematically retrieves, evaluates, and summarizes relevant evidence from both domestic and international sources, aiming to provide an evidence‑based foundation for developing an optimization plan for the caregiving behaviors of family caregivers of enterostomy patients.

## 2. Methods

This study is an evidence summary designed to systematically identify, appraise, and synthesize the best available evidence on optimization strategies for caregiving behaviors of family caregivers of enterostomy patients. While it adopts the rigorous methodological framework of systematic reviews—including a comprehensive search, predefined eligibility criteria, independent quality appraisal, and PRISMA-guided reporting—it does not perform quantitative meta-analysis, as the goal is to produce a practice-oriented evidence synthesis rather than a pooled effect estimate.The study protocol was prospectively registered with the Evidence‑Based Nursing Center of Fudan University (Registration No. ES20259473). The search and screening process adhered to the Preferred Reporting Items for Systematic Reviews and Meta‑Analyses (PRISMA) checklist (S1 File). Institutional review board approval and participant informed consent were not required because this study synthesized data from previously published literature.

### 2.1. Problem formulation

An evidence‑based question was formulated using the PIPOST model [9]. The target population (P) was family caregivers of enterostomy patients; the intervention (I) consisted of comprehensive support strategies including assessment, education, skill training, psychological support, and resource linkage for caregivers; the professionals (P) involved were healthcare professionals; the main outcomes (O) were caregivers’ caregiving ability and burden and patients’ quality of life; the settings (S) included hospitals, communities, and families; and the types of evidence (T) included clinical guidelines, systematic reviews, evidence summaries, expert consensuses, and randomized controlled trials.

### 2.2. Search strategy

Following the updated “5S” evidence pyramid model (2016) [10,11], a top‑down search was conducted in domestic and international databases, including UpToDate, BMJ Best Practice, GIN (Guidelines International Network), AHRQ (Agency for Healthcare Research and Quality), RNAO (Registered Nurses’ Association of Ontario), NICE (National Institute for Health and Care Excellence), Yimaitong Guideline Network, WCET (World Council of Enterostomal Therapists), WOCN (Wound, Ostomy and Continence Nurses Society), Chinese Medical Journal Database, Cochrane Library, Embase, PubMed, CINAHL, CBM (Chinese Biomedical Literature Database), CNKI, WanFang Data, and VIP, to identify evidence on behavior optimization strategies for family caregivers of elderly enterostomy patients.

English search terms included: “ostomy/stoma/enterostomy/intestinal stoma/colon stoma”, caregiver/care giver/carer/family caregiver/spouse caregiver/informal caregiver”, “intervention/program/support/education”. Studies on family caregivers of children with enterostomy were excluded. The search period covered from database inception to January 31, 2026. The complete search syntax for all databases, including all MeSH terms, Boolean operators, and final search combinations, is provided in S2 File.

### 2.3. Literature inclusion and exclusion criteria

Inclusion criteria: The research subjects are family caregivers of patients with enterostomy; the research content includes articles on enterostomy care, caregivers’ knowledge, skills, burden, health education, etc.; the research types include clinical practice guidelines, systematic reviews/Meta-analyses, evidence summaries, expert consensuses, and randomized controlled trials.

Exclusion criteria: Duplicate literatures; research types that are only abstracts or research design documents; literatures with incomplete reported data or those for which the full-text cannot be obtained.

**Fig 1:**
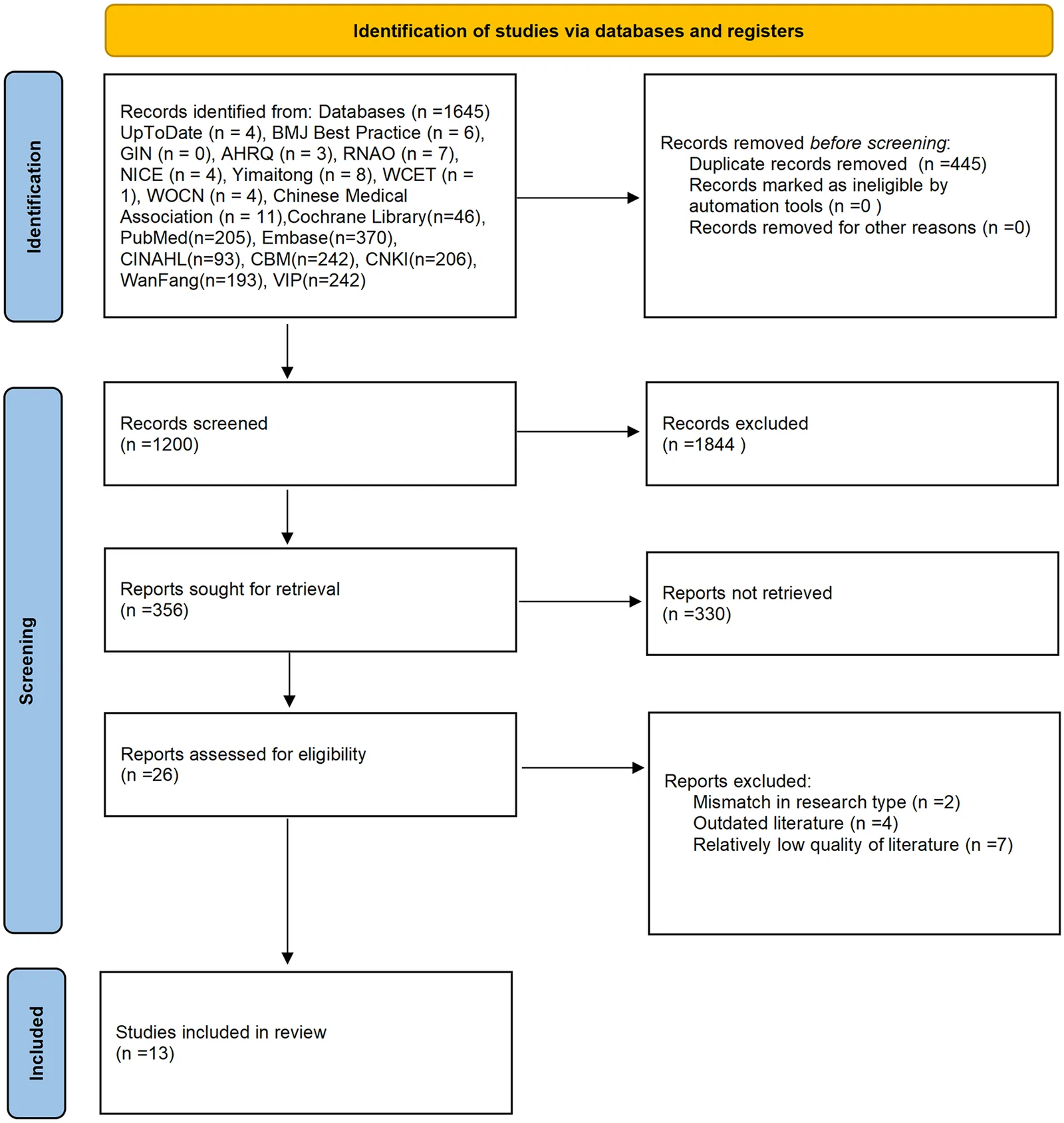
PRISMA 2020 flow diagram of study selection.

Study selection was conducted independently by two reviewers (Y.L. and Y.Z.) who had received systematic evidence-based method training. They first screened titles and abstracts against the eligibility criteria. Full texts of potentially eligible records were then retrieved and independently assessed by the same two reviewers. Disagreements at any stage were resolved through discussion or by consultation with a third reviewer (A.L.). The reasons for exclusion at the full-text stage were recorded and are presented in the PRISMA flow diagram (Fig 1).

### 2.4. Literature quality evaluation

In this study, corresponding evaluation criteria were adopted for different types of literature. For clinical decision-making literature: Since it integrates high-quality evidence and has been rigorously verified, it was directly included in the study without further evaluation. For clinical guidelines: The internationally recognized AGREE Ⅱ tool [12] was used to systematically evaluate the quality of the guidelines from multiple dimensions. For systematic reviews, expert consensuses, randomized controlled trials, and quasi-experimental studies: The corresponding evaluation forms of the JBI Evidence-Based Healthcare Center [13] were used for quality evaluation. This tool has specific standards for different research types to ensure the reliability and applicability of the evidence.

### 2.5. Literature quality evaluation process

Literature quality evaluation was independently performed by at least two researchers who had received systematic evidence‑based method training. The JBI Evidence Pre‑grading and Recommendation System (2014 version) [14] was used to grade the extracted evidence. Evidence was classified into levels 1–5 according to study design. In case of disagreement, a third researcher with relevant experience was consulted to reach consensus. If conflicting evidence conclusions existed, the principles of “evidence‑based priority, high‑quality priority, and latest publication priority” were followed.

When the extracted evidence required contextual interpretation (e.g., applicability to different healthcare settings or cultural contexts), professional opinions were incorporated through a structured process: the research team, which included two clinical specialists in enterostomy care from the gastrointestinal surgery department, reviewed the evidence items and provided feedback on their feasibility and relevance in local clinical practice. Their input was used to refine the wording and recommendations of the evidence items, particularly regarding implementation strategies. This process was documented and reviewed by all authors to ensure transparency.

## 3. Results

### 3.1. Literature screening process and basic information of included studies

A total of 1,645 potentially relevant records were identified from the initial search. After removing duplicates using EndNote, 1,200 records remained. Screening by title and abstract excluded 844 obviously irrelevant records. The full texts of the remaining 356 records were assessed, and 330 were excluded, leaving 26 records. Ultimately, 13 publications met the inclusion criteria: 2 clinical decisions [15,16], 8 guidelines [17–24], 1 systematic review [25], and 2 expert consensuses [26,27]. The literature screening process is shown in Fig 1. The basic characteristics of the included publications are presented in Table 1.

**Table 1:** Sources and basic information of evidence.

Included literature	Publication year	Literature source	Literature type	Literature theme
Ron G Landmann et al. [15]	2024	UpToDate	Clinical decision-making	Ileostomy or colostomy care and complications
Todd D Francone [16]	2024	UpToDate	Clinical decision-making	Overview of surgical ostomy for fecal diversion
Registered Nurses’ Association of Ontario [17]	2019	RNAO	Guideline	Supporting Adults Who Anticipate or Live with an Ostomy
Registered Nurses’ Association of Ontario [18]	2023	RNAO	Guideline	Association of Ontario.Caring for a Person with an Ostomy
Registered Nurses’ Association of Ontario [19]	2025	RNAO	Guideline	Person-centered care
NICE [20]	2020	NICE	Guideline	Supporting adult carers
Chinese Medical Association [21]	2025	Yimaitong	Guideline	Adult enterostomy care
American Society of Colon and Rectal Surgeons [22]	2022	Yimaitong	Guideline	Ostomy Surgery
Wound, Ostomy and Continence Nurses Society et al. [23]	2018	WOCN	Guideline	Management of adult patients with fecal or urinary stomas
World Council of Enterostomal Therapists [24]	2021	WCET	Guideline	International ostomy guideline
Heydari A et al. [25]	2023	PubMed	Systematic Review	Nursing Intervention for Quality of Life in Patients with Ostomy
Colorectal Surgery Group of the Surgery Branch of Chinese Medical Association et al. [26]	2025	Chinese Medical Association	Expert consensus	Permanent stoma in colorectal (cancer) surgery
Ratliff CR et al. [27]	2021	WOCN	Expert consensus	Peristomal skin health

### 3.2. Results of literature quality evaluation

#### 3.2.1. Quality evaluation of included clinical decisions and guidelines.

The two clinical decisions [15,16] were directly included without further evaluation because they integrate high‑quality evidence and have been strictly verified. The eight guidelines [17–24] were evaluated using the AGREE II tool. The scores in all six domains were ≥60%, and all guidelines were rated as grade A. The evaluation results are shown in Table 2.

**Table 2:** Methodological quality evaluation of the guidelines included in this study.

Included literature	Standardized percentage in each domain (%)						Overall quality score of the guideline in the comprehensive evaluation	Comprehensive evaluation recommends using the guideline	Recommendation level
	Scope and purpose	Participants	Rigor of development	Clarity of presentation	Applicability of the guideline	Editorial independence			
Registered Nurses’ Association of Ontario [17]	100	91.67	100	88.54	87.5	100	6.5	6.75	A
Registered Nurses’ Association of Ontario [18]	98.61	80.56	68.06	93.75	87.5	72.92	5.75	6	A
Registered Nurses’ Association of Ontario [19]	98.61	97.22	82.87	96.88	89.58	100	6.75	6.75	A
NICE [20]	100	75	72.69	98.96	83.33	70.83	6	5.75	A
Chinese Medical Association [21]	100	83.33	79.63	88.54	66.67	100	5.75	5.75	A
American Society of Colon and Rectal Surgeons [22]	100	69.44	95.37	70.83	66.67	100	5.75	5.75	A
Wound, Ostomy and Continence Nurses Society [23]	100	88.89	87.04	88.54	66.67	91.67	6.5	6.5	A
World Council of Enterostomal Therapists [24]	97.22	94.44	83.80	72.92	77.08	100	5.75	5.75	A

#### 3.2.2. Quality assessment of the included systematic reviews.

The one systematic review [25] was evaluated using the JBI critical appraisal checklist for systematic reviews. Except for item 9 (“Was the possibility of publication bias assessed?”), which was rated “No”, all other items were rated “Yes”, indicating relatively high overall quality.

#### 3.2.3. Quality evaluation of included expert consensuses.

The two expert consensuses [26,27] were evaluated using the JBI critical appraisal checklist for expert consensus articles. All six items were rated “Yes” for both consensuses, resulting in a quality grade of A. The evaluation results are detailed in Table 3.

**Table 3:** Methodological quality evaluation of expert consensus included in this study.

Included literature	①	②	③	④	⑤	⑥	Quality grade
Colorectal Surgery Group of the Surgery Branch of Chinese Medical Association et al. [26]	Yes	Yes	Yes	Yes	Yes	Yes	A
Ratliff CR et al. [27]	Yes	Yes	Yes	Yes	Yes	Yes	A

Note: ① Is the source of opinions clearly stated? ② Are the opinions from influential experts in the field? ③ Are the opinions centered on the interests of the target population? ④ Are the conclusions based on analysis results? Is the opinion expression logical? ⑤ Are other literature referenced and accurately cited? ⑥ Do the opinions differ from previous literature?

### 3.3. Summary results of evidence

After organizing and extracting the evidence, a total of 16 evidence items were summarized across four domains: structured empowerment of care knowledge and skills, support strategies for the care process, maintenance of caregivers’ physical and mental health, and construction of a multi‑dimensional support system. See Table 4 for details.

**Table 4:** Summary table of evidence.

Evidence Category	Evidence Content	Evidence Level
Structured empowerment of care knowledge and skills	1. Standardized training for core care skills: Enterostomal therapists should provide face-to-face and structured skill training to caregivers. The training content includes: ostomy bag replacement (disassembly, cleaning, measurement, and pasting), skin care (use of barrier paste/powder), identification and management of leakage, and methods for odor and gas control. The training should combine operational demonstrations with simulated exercises, usually arranged in 2–3 sessions, each lasting 40–60 minutes [15,21,22,25].	1
	2. Complication identification and emergency education: Caregivers should be able to recognize the early signs of common complications, such as dehydration (oliguria, dry mouth, fatigue), peristomal irritant dermatitis (redness, erosion), stoma prolapse/ischemia (darkening of color, swelling), high output (>1500–2000 ml/d), and know the basic coping measures and clear indications for seeking medical attention [15,17,21,23].	2
	3. Comprehensive management ability of peristomal skin health: Caregivers should learn to assess the skin health status and master key nursing operations: gently remove the stoma baseplate (at a low angle and support the skin), clean with clean water (avoid alkaline soap), shave the hair in the direction of hair growth, and select barrier products with reliable adhesion and low peeling force (such as a barrier containing ceramides) [27].	5
	4. Guidance on nutrition and fluid management: It is necessary to master the fluid replacement targets for preventing dehydration (especially for ileostomy) (an additional 500–750 ml per day), identify “obstruction-causing foods” (such as mushrooms, corn, celery), understand the list of gas-producing/odor-causing foods, assist with dietary adjustment, and use soluble fiber supplements or oral rehydration salts when there is high output [15,26].	2
	5. Guidance on the use of standardized assessment tools: Under the guidance of professionals, learn to use standardized assessment tools (such as the stoma skin tool DET score and the peristomal skin assessment tool) to objectively describe and monitor the condition of the stoma and the surrounding skin for accurate communication of problems [18,27].	5
Support strategies for the care process	6. Participate in the formulation of individualized care plans: Caregivers should jointly develop care plans with professionals. Based on the type of stoma, abdominal contour, and the patient’s self-care ability, select appropriate products (one-piece/two-piece, flat/convex) and learn to use auxiliary products (leak-proof rings, belts) [15,23,24].	2
	7. Care behavior assistance technologies and tools: Multimedia education packages (videos, illustrated manuals) and stoma care models can be used for simulation exercises. Encourage the use of remote support tools (telephone, applications) for problem consultation, and keep a care diary for communication during follow-up [17,24,25].	1
	8. Guidance on the adaptation of home environment and lifestyle: Lifestyle guidance such as methods for adjusting the home environment (e.g., storage, cleaning), skills for clothing selection and ostomy bag concealment, and precautions during bathing, traveling, and exercising should be provided to improve the convenience of care and the quality of life of patients [15].	5
Maintenance of the physical and mental health of caregivers	9. Preoperative emotional support and shared decision-making: Caregivers should participate in preoperative education, including discussions on stoma location, explanations of surgical expectations, and psychological counseling, to reduce anxiety in both parties and improve post-operative collaborative ability [16].	1
	10. Comprehensive needs assessment and risk identification for caregivers: Healthcare professionals should actively and regularly assess the needs and burdens of caregivers from multiple dimensions including physiology, psychology, society, economy, and environment, and identify risks such as “implicit bias” and “compassion fatigue” [19,20].	5
	11. Psychological support and stress management: Individuals should receive psychological education and learn personal stress management techniques (such as progressive muscle relaxation training). Meanwhile, they can express their emotions and obtain external emotional support by participating in motivational interviews or support groups. In addition, a communication support framework should be provided to improve the communication and decision-making patterns within the family and relieve relational stress at the systemic level [19,20,25].	1
	12. Respite care and alternative care: One should be aware of and have the right to access “respite care”, which allows oneself to take a break through short-term alternative care. Families are encouraged to divide care-giving tasks among family members [20].	4
Construction of a multi-dimensional support system	13. Collaborative support from professionals: Medical institutions should ensure that healthcare professionals receive systematic training, follow evidence-based guidelines, and implement person-centered and culturally sensitive nursing practices to support the optimization of caregivers’ care behaviors [24].	1
	14. Multidisciplinary team support and clear roles: Caregivers should be recognized as “expert members of the nursing team” and communicate effectively with stoma therapists, doctors, nurses, nutritionists, psychologists, etc., and clarify their respective roles [17,20,24,26].	4
	15. Structured follow-up and continuous care pathway: Caregivers should be included in the structured follow-up plan from the hospital to the home (such as the first follow-up within 1–2 weeks after discharge) to ensure that care-related problems can receive continuous professional answers and support [17,22,23].	1
	16. Social, economic, and information resource support: Clear guidance and resource links regarding social support (such as community services, peer support groups), economic assistance, legal rights, and how to access reliable health information should be provided [20].	4

## 4. Discussion

### 4.1. Structured training of knowledge and skills is the core foundation for improving the quality of care

International studies report that the incidence of complications after enterostomy ranges from 21% to 71% [28], whereas Chinese studies report rates of 16.3% to 53.8% [29]. Knowledge and skills remain the core needs of family caregivers. The high consistency of this recommendation across guidelines is unsurprising: inadequate training directly leads to peristomal complications and unplanned readmissions, which are well-documented drivers of poor patient outcomes and increased healthcare costs. Evidence items 1‑5 summarize structured empowerment strategies, including standardized training in core care skills, education on complication recognition and emergency response, comprehensive management of peristomal skin health, guidance on nutrition and fluid management, and instruction on using standardized assessment tools. Guidelines recommend that stoma specialist nurses provide face‑to‑face, structured training for caregivers, usually arranging 2‑3 sessions, each lasting 40‑60 minutes [25]. The specificity of these procedural recommendations contrasts sharply with the more general guidance on nutrition and fluid management. This discrepancy likely reflects that procedural skills are easily standardized and assessed, whereas dietary education inherently requires individualization based on stoma type, patient preferences, and cultural food practices—explaining why guidelines defer to general principles rather than prescriptive lists. However, in China, stoma specialist nurses also undertake other clinical duties, resulting in heavy workloads that make it difficult to implement structured caregiver empowerment [30]. Additionally, under enhanced recovery after surgery protocols, hospital stays are shortened, and caregivers often struggle to fully master the required knowledge and skills during hospitalization. This tension—between the time required for adequate training and the shrinking window of hospitalization—reveals a structural constraint that cannot be resolved by simply adding more in‑hospital sessions. It points to the need for alternative models, such as pre‑admission education or post‑discharge reinforcement, rather than relying solely on inpatient teaching. Most current training still focuses on short‑term skill teaching, lacking follow‑up support for long‑term skill application and behavior maintenance, and training formats have not fully adapted to individual differences and family situations.Standard stoma bag replacement and peristomal skin care are basic requirements for preventing complications. At the same time, using standardized assessment tools and timely identification and treatment are key measures to improve complication outcomes [18,27]. Nutrition and fluid management are easily overlooked aspects; studies have shown that effective nutritional support and fluid management can reduce the incidence of stoma complications [15,26,31]. As informal caregivers of enterostomy patients in home care, empowering family caregivers is beneficial for improving patients’ quality of life. In China, as in many other countries, the implementation of structured caregiver empowerment is further constrained by the competing clinical demands placed on stoma specialist nurses, who are often responsible for direct patient care alongside educational duties [30]. Future efforts should develop flexible, accessible training models and explore empowerment strategies suitable for different cultural backgrounds and educational levels to establish a more adaptive and sustainable support system.

### 4.2. Targeted process support strategies can effectively improve the caregiving ability of caregivers

The home environment has become an important setting for the rehabilitation of enterostomy patients. Evidence items 6‑8[15,17,23‑25] summarize support strategies for the care process. The evidence shows that jointly formulating care plans, using assistive technologies and tools, keeping care diaries, and providing lifestyle guidance can effectively improve caregivers’ caregiving ability and coping level, and enhance caregiving behaviors. The common thread across these strategies is a shift from passive instruction to active partnership—caregivers who participate in planning and documentation develop ownership that enhances both skill retention and problem-solving ability. However, some caregivers have difficulty benefiting due to insufficient digital skills or poor resource accessibility, which is related to age, educational background, and economic conditions. This digital divide raises concerns about equity of access: without targeted adaptations, the very technologies designed to support caregiving risk exacerbating existing disparities, particularly because these populations may bear the greatest caregiving burdens. Future interventions should consider the needs of this population and explore more convenient methods [32]. Care diaries can accurately record problems for professional consultation, but currently no standard format or feedback mechanism exists for care diaries, reducing their value for communication and guidance. A structured, validated diary format would not only guide caregivers’ observations but also facilitate professional interpretation and timely intervention, yet its development and validation remain unexplored in the literature. Meanwhile, lifestyle guidance is mostly fragmented. Further integration of nursing knowledge with life skills, and strengthened promotion and validation in community and home settings, is needed to improve caregivers’ autonomy and adaptability in the actual care process.

### 4.3. Maintaining the physical and mental health of caregivers is a key link in improving the overall care effect

Long‑term caregiving can impose varying degrees of burden on family caregivers, including physical, psychological, and economic burdens. This may lead to poor, formalistic care behaviors in which caregivers barely complete superficial tasks and ignore patients’ physical and mental needs, eventually reducing the quality of life of both patients and caregivers [33,34]. Maintaining family caregivers’ physical and mental health is conducive to improving care behaviors. Evidence items 9‑12 summarize comprehensive needs assessment and risk identification, psychological support and stress management, respite services and alternative care, and preoperative emotional support and shared decision‑making. Yet despite this strong endorsement, the literature provides remarkably little guidance on how these interventions should be operationalized—what specific techniques, frequency, or intensity are most effective. Healthcare professionals should regard caregivers as care partners, include them in nursing plan formulation, and involve them in preoperative education. Emotional support should be added to preoperative education to reduce anxiety in both parties and improve postoperative collaboration [16]. Unlike skill training, which follows a clear instructional logic, psychological support is inherently relational, context‑sensitive, and difficult to manualize—which likely explains why guidelines endorse its importance but stop short of specifying content, frequency, or delivery models. Family caregivers should receive psychological education and learn stress management skills. They should be encouraged to participate in motivational interviews or support groups to express emotions and obtain emotional support [25]. In addition, a communication support framework should be provided to improve family communication and decision‑making patterns and relieve relational stress at the systemic level [19,20]. Implementing these recommendations requires a stepped‑care approach—offering universal screening and low‑intensity support for all caregivers, with escalation to more intensive interventions for those at higher risk of burnout. At the same time, family caregivers should understand “respite services” and be encouraged to divide care tasks among family members [20]. However, the uptake of respite services may also be influenced by cultural factors—such as guilt about burdening others or lack of trust in substitute care—that remain underexplored in the current evidence base.

### 4.4. Building an integrated support system is the fundamental guarantee for achieving continuity of care

Evidence items 13‑16 indicate that building a support system covering multidisciplinary collaboration, continuous follow‑up, and social resource integration is an important guarantee for improving care quality. Existing guidelines advocate regarding caregivers as “team experts” and providing support through professional training, standardized pathways, and resource links. This rhetorical recognition, however, remains aspirational in most settings—translating it into reality requires not only clear protocols but also a fundamental shift in how healthcare professionals perceive caregivers: from passive recipients of instructions to active partners in care. Meanwhile, healthcare professionals should implement person‑centered and culturally sensitive nursing practices to support the optimization of caregivers’ care behaviors [17,20,24,26]. However, in practice, problems such as low efficiency of multidisciplinary collaboration, poor connection between in‑hospital and out‑of‑hospital care, and scattered social resource information remain widespread, limiting the actual effectiveness of support systems [34]. These are not merely operational failures—they reflect deeper structural issues: fragmented healthcare financing, misaligned professional incentives, and the absence of shared care protocols that transcend individual disciplines. To address these issues, future efforts should improve team collaboration and communication mechanisms, establish information‑based support platforms to connect in‑hospital and out‑of‑hospital care, and integrate medical, community, and social resources to provide clear and convenient support navigation for caregivers[17,22‑23]. However, technology and training alone are insufficient—they must be accompanied by institutional commitment to integrated care models that are clinically sound and economically sustainable, including reimbursement mechanisms for caregiver education and telehealth follow-up, alongside systematic professional development to equip healthcare professionals for their role as caregiver supporters [24].

### 4.5. Evidence gaps and future research priorities

Several evidence gaps warrant mention. Most included evidence originates from high‑income countries, limiting applicability to resource‑constrained settings where healthcare infrastructure and caregiver resources differ substantially. In addition, while psychosocial support and respite care are consistently endorsed, the literature provides little operational guidance on their delivery—including specific techniques, frequency, or intensity. Future research should address these gaps through implementation studies in diverse settings and well‑designed trials evaluating specific intervention components.

### 4.6. Clinical implications and recommendations for practice

This evidence summary highlights one central clinical imperative: structured caregiver training must move from ad‑hoc, one‑time bedside teaching to a planned curriculum of 2‑3 sessions (40‑60 minutes each) covering core skills, complication recognition, and skin care. Beyond training, clinicians should treat caregivers as active care partners—routinely assessing their burden and psychological status, integrating them into the multidisciplinary team, and establishing structured follow‑up pathways bridging hospital and home care. When resources are limited, practitioners should prioritize high‑level evidence (Levels 1‑2) while adapting lower‑level evidence (Levels 4‑5) to local contexts. Above all, all recommendations must be implemented in a person‑centered, culturally sensitive manner that recognizes the diversity of caregiver needs, resources, and readiness.

## 5. Limitations

This study has several limitations that should be acknowledged. First, although we performed a systematic search, some grey literature or ongoing studies might not have been captured, introducing potential publication bias. Second, the quality of the included evidence varied across different types of publications; however, all included literature met the minimum quality criteria and were rated as acceptable for evidence synthesis. Third, the evidence summary focuses on general optimization strategies without detailed subgroup analyses based on different types of enterostomy or patient characteristics, which could be explored in future research. Fourth, the relatively limited number of included studies (n = 13) may constrain the generalizability of findings, particularly for specific subdomains where evidence was particularly sparse (e.g., psychological support strategies, long-term care maintenance). While this is inherent to evidence summary designs that prioritize high-quality sources over exhaustive inclusion, the findings should be interpreted with consideration of the evidence base’s breadth. Future primary studies are needed to strengthen the evidence base in underrepresented areas. Despite these limitations, the synthesized evidence provides a reliable and practical reference for clinical practice.

## 6. Conclusions

This study summarizes the best evidence for optimizing caregiving behaviors of family caregivers of patients with enterostomy, covering four aspects: structured empowerment of care knowledge and skills, support strategies for the care process, maintenance of caregivers’ physical and mental health, and construction of a multi‑dimensional support system. The evidence provides a scientifically sound and practical reference for healthcare professionals to develop intervention plans and implement evidence‑based practice. When applying the evidence, healthcare professionals should consider the specific context, patient and caregiver characteristics, and available resources to select the most appropriate strategies.

## Supporting information

S1 File PRISMA 2020 checklist. (DOCX)

S2 File Complete search strategies for all databases (PubMed, Embase, CINAHL, Cochrane Library, CBM, CNKI, WanFang, VIP, and guideline websites), including database‑specific controlled vocabulary, Boolean operators, and search execution dates. (DOCX)
