# Supplementary material for: Optimization strategies for care‑giving behaviors of family caregivers for patients with enterostomy: An evidence summary
Source: PLoS One. 2026 Sep 10;21(9):e0356569. doi: 10.1371/journal.pone.0356569 (PMC13561317; doi:10.1371/journal.pone.0356569)
Supplement: Supplementary file 2 [file pone.0356569.s002.docx]

**Supplementary File 2. Complete Search Strategies for All Databases**

This file contains the full search syntax for all databases and websites used in this evidence summary. Searches were conducted from database inception to January 31, 2026. No language or date restrictions were applied. Pediatric populations (newborns, children, adolescents) were excluded.

For guideline websites and professional organizations (UpToDate, BMJ Best Practice, GIN, AHRQ, RNAO, NICE, Yimaitong, WCET, WOCN, Chinese Medical Association), the same search concepts were applied using free-text keyword combinations of "ostomy/stoma/enterostomy" AND "caregiver/carer". Results were manually screened against eligibility criteria.

**Note on Chinese database searches:** The Chinese search terms used in the CBM, CNKI, WanFang, and VIP databases correspond to the following English concepts: “肠造口/回肠造口/结肠造口/人工肛门” = enterostomy/ileostomy/colostomy; “家庭照顾者/主要照顾者/家属/照顾者/家庭照护者” = family caregivers/primary caregivers; “干预/方案/支持/教育” = intervention/program/support/education; “新生儿/小儿/儿童/患儿” = neonates/children/pediatric populations. The search syntax itself must remain in Chinese as required by the respective database interfaces.

**No.1 Cochrane library**

| **Search number** | **Query** |
| --- | --- |
| #1 | MeSH descriptor: [Ostomy] explode all trees |
| #2 | (ostomy):ti,ab,kw OR (stoma):ti,ab,kw OR (enterostomy):ti,ab,kw OR (intestinal stoma):ti,ab,kw OR (colon stoma):ti,ab,kw |
| #3 | #1 OR #2 |
| #4 | MeSH descriptor: [Caregivers] explode all trees |
| #5 | (caregiver):ti,ab,kw OR (carer):ti,ab,kw OR (family caregiver):ti,ab,kw OR (spouse caregiver):ti,ab,kw OR (informal caregiver):ti,ab,kw |
| #6 | #4 OR #5 |
| #7 | (intervention):ti,ab,kw OR (program):ti,ab,kw OR (support):ti,ab,kw OR (education):ti,ab,kw |
| #8 | #3 AND #6 AND #7 |
| #9 | (newborns):ti,ab,kw OR (children):ti,ab,kw OR (pediatrics):ti,ab,kw OR (pediatric patients):ti,ab,kw |
| #10 | #8 NOT #9 |

**No.2 PubMed**

| **Search number** | **Query** |
| --- | --- |
| #1 | ostomy[MeSH Terms] |
| #2 | ((((ostomy[Title/Abstract]) OR (stoma[Title/Abstract])) OR (enterostomy[Title/Abstract])) OR (intestinal stoma[Title/Abstract])) OR (colon stoma[Title/Abstract]) |
| #3 | #1 OR #2 |
| #4 | caregivers[MeSH Terms] |
| #5 | ((((((((caregiver*[Title/Abstract]) OR (care giver*[Title/Abstract])) OR (carer*[Title/Abstract])) OR (family caregiver*[Title/Abstract])) OR (caregiver*, family[Title/Abstract])) OR (spouse caregiver*[Title/Abstract])) OR (caregiver*, spouse[Title/Abstract])) OR (informal caregiver*[Title/Abstract])) OR (caregiver*, informal[Title/Abstract]) |
| #6 | #4 OR #5 |
| #7 | (((intervention[Title/Abstract]) OR (program[Title/Abstract])) OR (support[Title/Abstract])) OR (education[Title/Abstract]) |
| #8 | #3 AND #6 AND #7 |
| #9 | (((newborns[Title/Abstract]) OR (children[Title/Abstract])) OR (pediatrics[Title/Abstract])) OR (pediatric patients[Title/Abstract]) |
| #10 | #8 NOT #9 |

**No.3 Embase**

| **No.** | **Query Results** | **Results** | **Date** |
| --- | --- | --- | --- |
| #1. | 'stoma'/exp OR 'stoma' | 32,523 | 31 Jan 2026 |
| #2. | 'ostomy'/exp OR 'ostomy' | 136,846 | 31 Jan 2026 |
| #3. | ostomy:ti,ab,kw OR stoma:ti,ab,kw OR enterostomy:ti,ab,kw OR 'intestinal stoma':ti,ab,kw OR 'colon stoma':ti,ab,kw | 27,346 | 31 Jan 2026 |
| #4. | #1 OR #2 OR #3 | 158,748 | 31 Jan 2026 |
| #5. | 'caregiver'/exp OR 'caregiver' | 184,347 | 31 Jan 2026 |
| #6. | caregiver:ti,ab,kw OR carer:ti,ab,kw OR 'family caregiver':ti,ab,kw OR 'spouse caregiver':ti,ab,kw OR 'informal caregiver':ti,ab,kw | 92,999 | 31 Jan 2026 |
| #7. | #5 OR #6 | 190,742 | 31 Jan 2026 |
| #8. | intervention:ti,ab,kw OR program:ti,ab,kw OR support:ti,ab,kw OR education:ti,ab,kw | 4,695,972 | 31 Jan 2026 |
| #9. | #4 AND #7 AND #8 | 816 | 31 Jan 2026 |
| #10. | #4 AND #7 AND #8 AND ([adult]/lim OR [aged]/lim OR [very elderly]/lim) | 370 | 31 Jan 2026 |

**No.4 CINAHL**

Search syntax:

XB (ostomy OR stoma OR enterostomy OR intestinal stoma OR colon stoma) AND XB (caregiver OR care giver OR carer OR family caregiver OR spouse caregiver OR informal caregiver) AND XB (intervention OR program OR support OR education)

**No.5 CNKI (China National Knowledge Infrastructure)**

Search syntax:

(SU="肠造口" OR SU="回肠造口" OR SU="结肠造口" OR SU="人工肛门") AND (SU="家庭照顾者" OR SU="主要照顾者" OR SU="家属" OR SU="照顾者" OR SU="家庭照护者") AND (SU="干预" OR SU="方案" OR SU="支持" OR SU="教育") NOT (SU="新生儿" OR SU="小儿" OR SU="儿童" OR SU="患儿")`

**No.6 WanFang Data**

Search syntax:

((主题:(肠造口) OR 题名或关键词:(回肠造口) OR 题名或关键词:(结肠造口) OR 题名或关键词:(人工肛门)) AND (主题:(家庭照顾者) OR 题名或关键词:(主要照顾者) OR 题名或关键词:(家属) OR 题名或关键词:(照顾者) OR 题名或关键词:(家庭照护者)) AND (题名或关键词:(干预) OR 题名或关键词:(方案) OR 题名或关键词:(支持) OR 题名或关键词:(教育))) NOT (题名或关键词:(新生儿) OR 题名或关键词:(小儿) OR 题名或关键词:(儿童) OR 题名或关键词:(患儿))

**No.7 VIP (Chinese Science and Technology Journal Database)**

Search syntax:

(((M=肠造口 OR R=回肠造口) OR R=结肠造口) OR R=人工肛门) AND ((((M=家庭照顾者 OR R=主要照顾者) OR R=家属) OR R=照顾者) OR R=家庭照护者) AND (((R=干预 OR R=方案) OR R=支持) OR R=教育) NOT (((R=新生儿 OR R=小儿) OR R=儿童) OR R=患儿)

**No.8 CBM (Chinese Biomedical Literature Database)**

| **Search number** | **Query Results** | **Results** | **Date** |
| --- | --- | --- | --- |
| 1) | "肠造口"[Intelligent Field] OR "回肠造口"[Intelligent Field] OR "结肠造口"[Intelligent Field] OR "人工肛门"[Intelligent Field] | 11057 | 2026-01-31 17:46:45.0 |
| 2) | "家庭照顾者"[Intelligent Field] OR "主要照顾者"[Intelligent Field] OR "家属"[Intelligent Field] OR "照顾者"[Intelligent Field] OR "家庭照护者"[Intelligent Field] | 85415 | 2026-01-31 17:48:36.0 |
| 3) | "干预"[Intelligent Field] OR "方案"[Intelligent Field] OR "支持"[Intelligent Field] OR "教育"[Intelligent Field] | 1943602 | 2026-01-31 17:49:43.0 |
| 4) | (#3) AND (#2) AND (#1) | 293 | 2026-01-31 17:49:59.0 |
| 5) | "新生儿"[Intelligent Field] OR "小儿"[Intelligent Field] OR "儿童"[Intelligent Field] OR "患儿"[Intelligent Field] | 922451 | 2026-01-31 17:51:27.0 |
| 6) | ((#4) NOT (#5)) | 242 | 2026-01-31 17:53:53.0 |
